# Supplementary material for: A Pleiotropic and Functionally Divergent RAC3 Variant Disrupts Neurodevelopment and Impacts Organogenesis
Source: Cells. 2025 Sep 24;14(19):1499. doi: 10.3390/cells14191499 (PMC12523998; doi:10.3390/cells14191499)
Supplement: Supplementary file 1 [file cells-14-01499-s001.zip › cells-3781202-supplementary/cells-3781202-supplymentary/Suppl Figure S1.pptx]

## Slide 1
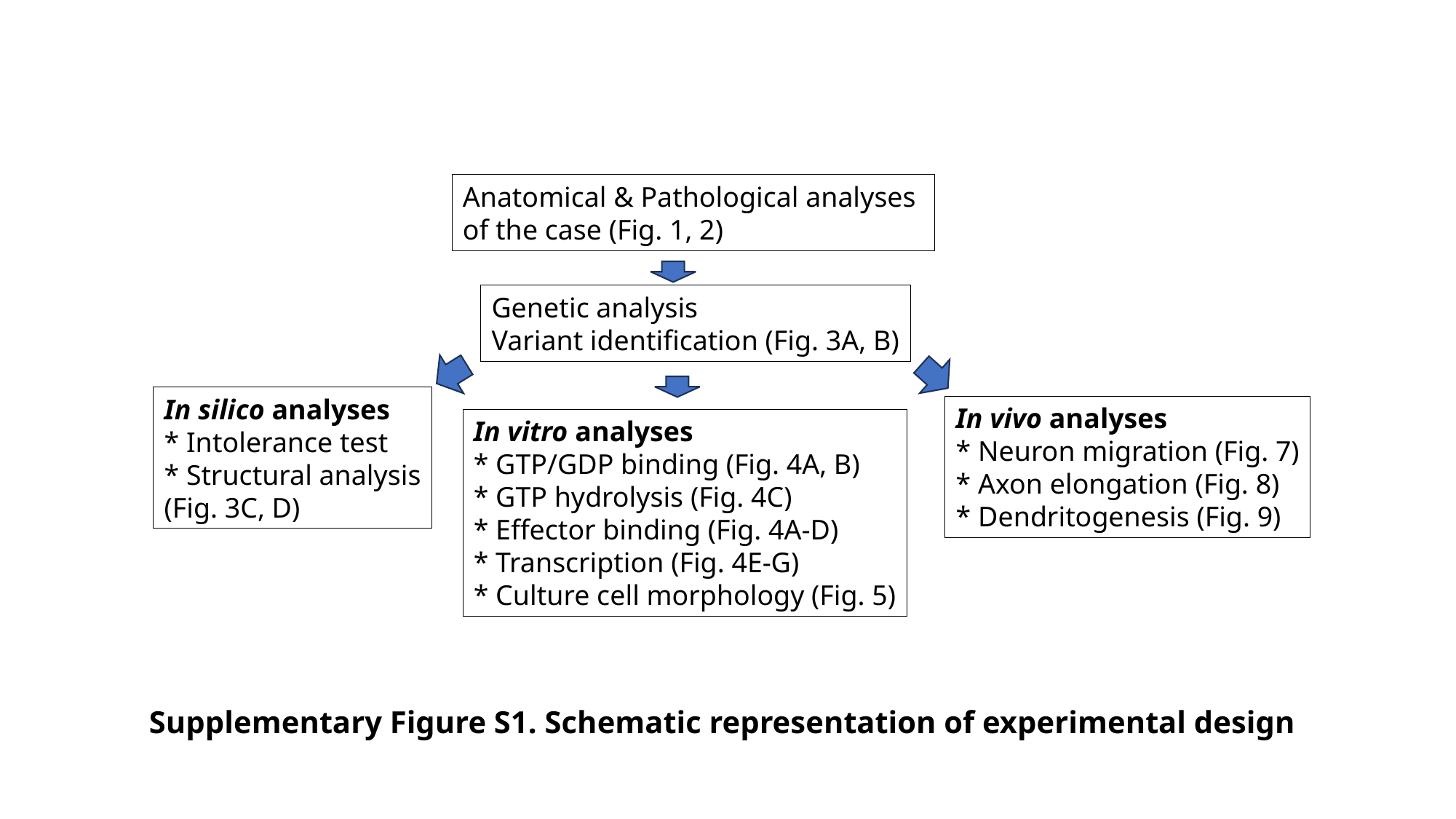

Anatomical & Pathological analyses
of the case (Fig. 1, 2)
Genetic analysis
Variant identification (Fig. 3A, B)
In silico analyses
* Intolerance test
* Structural analysis
(Fig. 3C, D)
In vivo analyses
* Neuron migration (Fig. 7)
* Axon elongation (Fig. 8)
* Dendritogenesis (Fig. 9)
In vitro analyses
* GTP/GDP binding (Fig. 4A, B)
* GTP hydrolysis (Fig. 4C)
* Effector binding (Fig. 4A-D)
* Transcription (Fig. 4E-G)
* Culture cell morphology (Fig. 5)
Supplementary Figure S1. Schematic representation of experimental design
