# Supplementary figures and images for: A Pleiotropic and Functionally Divergent RAC3 Variant Disrupts Neurodevelopment and Impacts Organogenesis
Source: Cells. 2025 Sep 24;14(19):1499. doi: 10.3390/cells14191499 (PMC12523998; doi:10.3390/cells14191499)

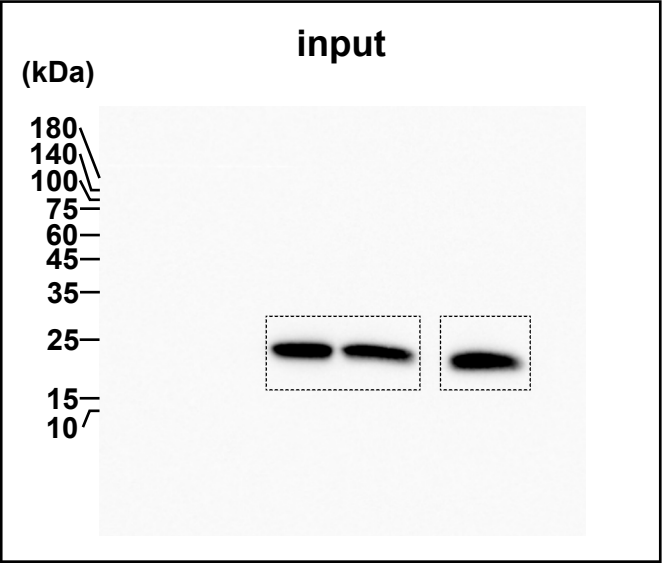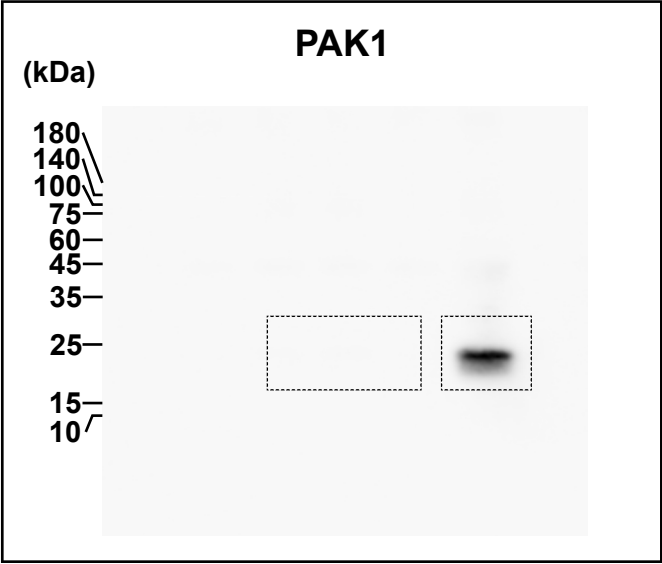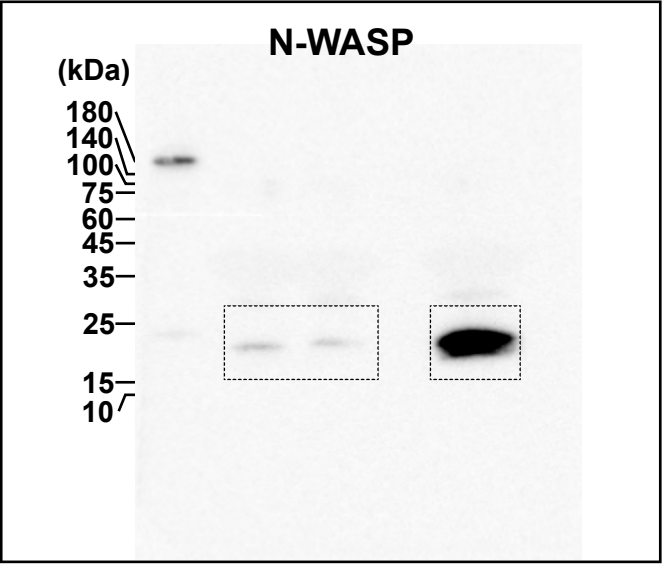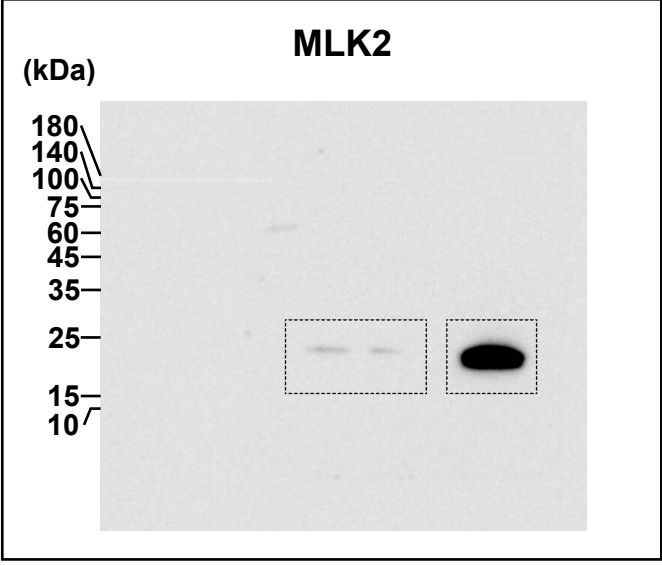

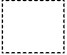 : Cropping line

Supplement: Supplementary file 1 [file cells-14-01499-s001.zip › cells-3781202-supplementary/cells-3781202-supplymentary/Suppl Figure S2.pdf]

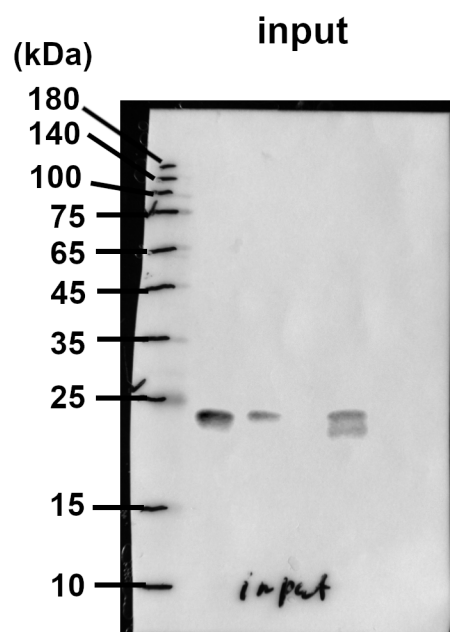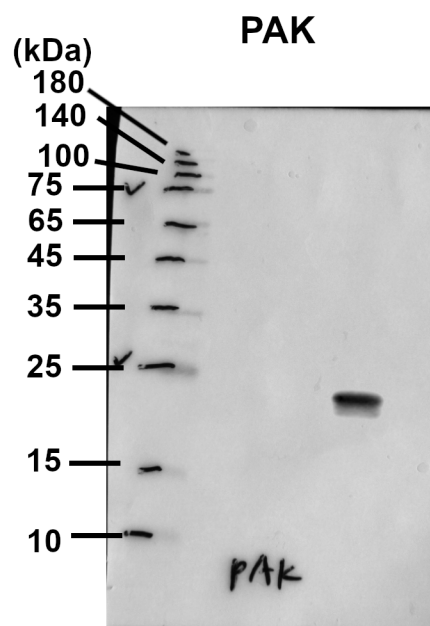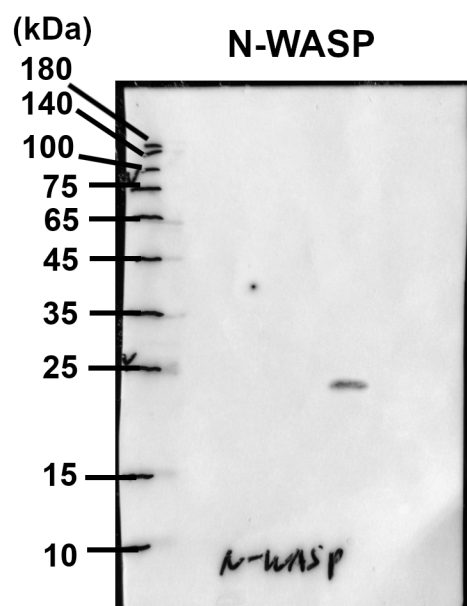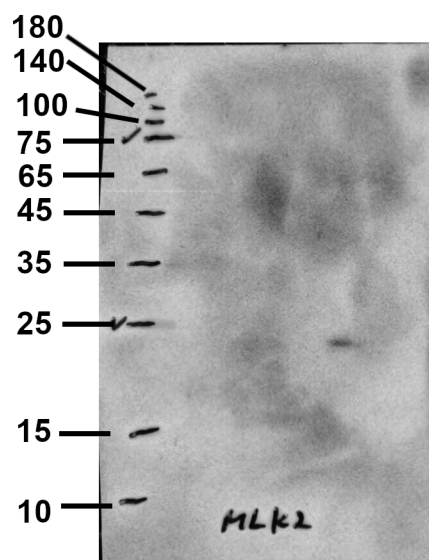

Supplement: Supplementary file 1 [file cells-14-01499-s001.zip › cells-3781202-supplementary/cells-3781202-supplymentary/Suppl Figure S3.pdf]
